# Correction: Dabi et al. Clues for Improving the Pathophysiology Knowledge for Endometriosis Using Plasma Micro-RNA Expression. *Diagnostics* 2022, *12*, 175

**DOI:** 10.3390/diagnostics14080829

**Published:** 2024-04-17

**Authors:** Yohann Dabi, Stéphane Suisse, Ludmila Jornea, Delphine Bouteiller, Cyril Touboul, Anne Puchar, Emile Daraï, Sofiane Bendifallah

**Affiliations:** 1 Department of Obstetrics and Reproductive Medicine, Hôpital Tenon, Sorbonne University, 4 Rue de la Chine, 75020 Paris, France; 2 Clinical Research Group (GRC) Paris 6, Centre Expert Endométriose (C3E), Sorbonne University (GRC6 C3E SU), 4 Rue de la Chine, 75020 Paris, France; 3 Cancer Biology and Therapeutics, Centre de Recherche Saint-Antoine (CRSA), Sorbonne University, INSERM UMR_S_938, 75020 Paris, France; 4 Ziwig Health, 19 Rue Reboud, 69003 Lyon, France; 5 Paris Brain Institute—Institut du Cerveau—ICM, Inserm U1127, CNRS UMR 7225, AP-HP—Hôpital Pitié-Salpêtrière, Sorbonne University, 4 Rue de la Chine, 75020 Paris, France; 6 Gentoyping and Sequencing Core Facility, iGenSeq, Institut du Cerveau et de la Moelle Épinière, ICM, Hôpital Pitié-Salpêtrière, 47–83 Boulevard de l’Hôpital, 75013 Paris, France

## Corrected Title

In the original publication [1], there was an error in the title of the original publication. The correct title is “Clues for Improving the Pathophysiology Knowledge for Endometriosis Using Plasma Micro-RNA” instead of “Serum Micro-RNA”.

## Corrected Figure Legend

There was a mistake in the legend for Figure 1: it was not serum but plasma that was used in our experiments. The correct legend appears below.

**Figure 1.** miRNA plasma expression according to endometriosis stage.

The authors state that the scientific conclusions are unaffected. This correction was approved by the Academic Editor. The original publication has also been updated.
