# Supplementary material for: Clues for Improving the Pathophysiology Knowledge for Endometriosis Using Plasma Micro-RNA Expression
Source: Diagnostics (Basel). 2022 Jan 12;12(1):175. doi: 10.3390/diagnostics12010175 (PMC8774370; doi:10.3390/diagnostics12010175)
Supplement: Supplementary file 1 [file diagnostics-12-00175-s001.zip › Table S1.pdf]

Table S1. Distribution of miRNAs according to the AUC values.

**miRNAs with an AUC <0.5**	**miRNAs with an AUC ≥0.5 and <0.6**	**miRNAs with an AUC ≥0.6**
miR-346	miR-4524b-5p	miR-6502-5p
miR-7156-5p	miR-3122	miR-515-5p
miR-224-5p	miR-4715-5p	miR-548j-5p
miR-515-3p	miR-4712-3p	miR-29b-1-5p
miR-568	miR-4536-5p	miR-4748
miR-2392	miR-370-5p	miR-548p
miR-6090	miR-6074	miR-5697
miR-1299	miR-6805-3p	miR-3124-5p
miR-4535	miR-6726-5p	miR-4999-5p
miR-3139	miR-4436b-3p	miR-6501-5p
miR-34b-3p	miR-6794-5p	miR-1270
miR-9-5p	miR-7106-5p	miR-433-3p
miR-432-5p	miR-6788-5p	miR-548ah-3p
miR-4257	miR-6731-3p	miR-1278
miR-1237-3p	miR-2681-3p	miR-548l
miR-1281	miR-7852-3p	miR-4511
miR-3609	miR-6890-5p	miR-3940-3p
miR-1914-5p	miR-7113-3p	miR-5009-5p
miR-8075	miR-10397-5p	miR-10399-5p
miR-1261	miR-4531	miR-1292-5p
miR-4670-3p	miR-5698	miR-144-5p
miR-409-3p	miR-6819-3p	miR-3942-5p
miR-1229-3p	miR-513b-3p	miR-92b-5p
miR-1469	miR-376c-5p	miR-362-5p
miR-5100	miR-3907	miR-1285-3p
miR-184	miR-3619-3p	miR-3913-5p
miR-3912-5p	miR-8086	miR-548q
miR-1290	miR-3128	miR-30e-3p
miR-4788	miR-6722-3p	miR-151a-3p
miR-211-5p	miR-100-3p	miR-4732-3p
miR-4656	miR-210-5p	miR-421
miR-372-3p	miR-4783-3p	miR-6789-5p
miR-1471	miR-487a-3p	miR-27b-5p
miR-4422	miR-548av-5p	miR-1910-3p
miR-3927-5p	miR-4477b	miR-6773-5p
miR-4662a-3p	miR-410-5p	miR-542-5p
miR-16-1-3p	miR-4765	miR-548f-5p
miR-561-5p	miR-4701-3p	miR-1250-5p
miR-495-5p	miR-6847-3p	miR-1972
miR-8063	miR-3135b	miR-548ay-3p

miR-494-3p
miR-502-5p
miR-6765-5p
miR-330-3p
miR-4999-3p
miR-4488
miR-4657
miR-142-3p
miR-376c-3p
miR-450a-2-3p
miR-6731-5p
miR-370-3p
miR-26a-1-3p
miR-6767-5p
miR-3914
miR-4665-5p
miR-6505-5p
miR-4526
miR-4741
miR-133a-3p
miR-369-3p
miR-320c
miR-548aq-5p
miR-4264
miR-6515-3p
miR-378h
miR-6852-5p
miR-215-5p
miR-653-3p
miR-146b-5p
miR-320b
miR-6753-5p
miR-320d
miR-1301-3p
miR-4687-5p
miR-5690
miR-6802-3p
miR-6765-3p
miR-670-5p
miR-4449
miR-219b-5p
miR-6089
miR-4469
miR-1286
miR-5688
miR-4726-5p
miR-3180-5p
miR-1256
miR-4647
miR-411-5p
miR-3140-5p
miR-4642
miR-520d-3p
miR-6795-5p
miR-676-5p
miR-380-5p
miR-181b-3p
miR-4747-3p
miR-3664-5p
miR-605-3p
miR-4724-5p
miR-6879-5p
miR-3059-3p
miR-376a-5p
miR-6790-5p
miR-4639-3p
miR-943
miR-548as-5p
miR-4474-3p
miR-4679
miR-5192
miR-4536-3p
miR-4688
miR-6082
miR-6865-5p
miR-6817-5p
miR-6513-5p
miR-548as-3p
miR-6787-3p
miR-4703-3p
miR-4719
miR-887-5p
miR-4424
miR-6860
miR-4502
miR-3617-5p
miR-4466
miR-6785-5p
miR-6777-5p
miR-4514
miR-6802-5p
miR-4658
miR-124-3p
miR-4655-5p
miR-1343-5p
miR-1266-5p
miR-548b-3p
miR-6509-5p
miR-7107-5p
miR-8089
miR-6813-5p
miR-3137
miR-3168

miR-3175
miR-6818-5p
miR-4286
miR-28-3p
miR-182-3p
miR-518c-5p
miR-135b-5p
miR-23a-5p
miR-6794-3p
miR-10398-3p
miR-6800-5p
miR-3164
miR-3665
miR-122b-5p
miR-33a-5p
miR-744-5p
miR-9903
miR-194-5p
miR-8076
miR-3938
let-7e-5p
miR-5693
miR-1911-5p
miR-490-5p
miR-483-5p
miR-642a-3p
miR-6799-5p
miR-155-5p
miR-1303
miR-545-5p
miR-577
miR-5588-5p
miR-382-5p
miR-34c-3p
miR-128-3p
miR-4433b-5p
miR-5684
miR-335-3p
miR-3678-3p
miR-517-5p
miR-6727-3p
miR-2278
miR-597-3p
miR-6761-3p
miR-4433a-3p
miR-376b-5p
miR-3156-5p
miR-3620-3p
miR-4779
miR-219a-5p
miR-4524a-5p
miR-659-3p
miR-6810-5p
miR-6855-3p
miR-9901
miR-525-5p
miR-146a-3p
miR-33b-3p
miR-541-3p
miR-6797-5p
miR-4638-3p
miR-616-5p
miR-1298-3p
miR-3129-5p
miR-4709-3p
miR-4290
miR-211-3p
miR-6132
miR-323a-5p
miR-4784
miR-4493
miR-3189-3p
miR-6869-3p
miR-6786-5p
miR-597-5p
miR-4430
miR-6825-3p
miR-1183
miR-551b-3p
miR-762
miR-135a-2-3p
miR-4518
miR-3193
miR-509-3-5p
miR-7111-5p
miR-4421

miR-3690
miR-371a-5p
miR-548e-5p
miR-3960
miR-548ad-5p
miR-548aj-5p
miR-532-3p
miR-548c-3p
miR-191-3p
miR-20a-5p
miR-3909
miR-4659b-3p
miR-1260a
miR-1284
miR-6837-3p
miR-99b-5p
miR-30e-5p
miR-19b-3p
miR-653-5p
miR-146a-5p
miR-139-3p
miR-6848-5p
miR-423-5p
miR-1273h-3p
miR-214-3p
miR-6791-5p
let-7d-3p
miR-548aq-3p
miR-328-3p
miR-584-5p
miR-150-3p
miR-3125
miR-345-5p
miR-5196-3p
miR-523-3p
miR-148a-3p
miR-505-3p
miR-502-3p
miR-6843-3p
miR-3130-5p
miR-4516
miR-543
miR-378d
miR-4525
miR-5007-5p
miR-503-3p
miR-6780b-3p
miR-4689
miR-11400
miR-600
miR-3155a
miR-4484
miR-203b-3p
miR-767-5p
miR-4649-5p
miR-548j-3p
miR-5001-5p
miR-4468
miR-6507-5p
miR-3180
miR-3180-3p
miR-517a-3p
miR-517b-3p
miR-4482-5p
miR-449a
miR-6072
miR-6853-3p
miR-12135
miR-4463
miR-7-2-3p
miR-4745-5p
miR-6506-5p
miR-4782-3p
miR-6083
miR-548ah-5p
miR-6893-3p
miR-921
miR-516b-5p
miR-12127
miR-7856-5p
miR-6755-5p
miR-4718
miR-6850-3p
miR-4534
miR-6763-3p
miR-892a

miR-548c-5p
miR-6842-5p
miR-6732-3p
miR-126-5p
miR-500a-3p
miR-30a-3p
miR-17-3p
miR-7847-3p
miR-651-5p
miR-3679-5p
miR-21-5p
miR-412-5p
miR-452-5p
miR-101-5p
miR-429
miR-186-5p
miR-1908-5p
miR-1307-3p
miR-6510-5p
miR-25-3p
miR-6741-5p
miR-23b-3p
miR-548g-5p
miR-381-3p
miR-361-3p
miR-378c
miR-4265
miR-200b-3p
miR-125a-3p
miR-664a-3p
miR-548am-5p
miR-3127-5p
miR-1322
miR-361-5p
miR-548o-5p
miR-10b-3p
miR-4742-3p
miR-570-3p
miR-4301
miR-146b-3p
miR-363-3p
miR-29c-3p
miR-127-5p

miR-6817-3p
miR-5187-3p
miR-1323
miR-605-5p
miR-3120-5p
miR-8069
miR-4271
miR-6812-3p
miR-196a-1-3p
miR-4701-5p
miR-3681-5p
miR-6769b-5p
miR-7855-5p
miR-4791
miR-367-5p
miR-5000-3p
miR-3622b-5p
miR-4786-3p
miR-493-3p
miR-4524a-3p
miR-6804-3p
miR-4681
miR-3187-5p
miR-6871-3p
miR-6774-3p
miR-379-5p
miR-1-5p
miR-6876-5p
miR-410-3p
miR-6856-3p
miR-6516-3p
miR-7853-5p
miR-6134
miR-6500-3p
miR-6753-3p
miR-5000-5p
miR-3171
miR-632
miR-6740-3p
miR-181d-3p
miR-490-3p
miR-5587-5p
miR-623

miR-1236-5p
miR-362-3p
miR-1285-5p
miR-29c-5p
miR-10a-3p
miR-489-3p
miR-450a-1-3p
miR-6883-5p
miR-339-3p
miR-342-5p
miR-4454
miR-30b-5p
miR-3065-5p
miR-196b-3p
miR-4708-3p
miR-29b-3p
miR-27b-3p
miR-154-5p
miR-769-5p
miR-548y
miR-676-3p
miR-181d-5p
miR-30a-5p
miR-221-3p
miR-518b
miR-136-3p
miR-4766-3p
miR-125a-5p
miR-6877-5p
miR-4496
miR-497-5p
miR-132-3p
miR-6809-5p
miR-3149
miR-27a-5p
miR-30c-1-3p
miR-223-3p
miR-122-5p
miR-153-3p
miR-455-5p
miR-1247-5p
miR-3200-5p
miR-181c-5p
miR-4313
miR-657
miR-4733-5p
miR-6085
miR-3617-3p
miR-6739-3p
miR-5088-5p
miR-6849-3p
miR-4434
miR-1237-5p
miR-4800-5p
miR-802
miR-1295a
miR-3659
miR-891a-3p
miR-6720-3p
miR-1909-3p
miR-561-3p
miR-302d-3p
miR-449b-5p
miR-6508-3p
miR-3612
miR-5195-5p
miR-7106-3p
miR-4538
miR-6730-5p
miR-588
miR-12136
miR-7114-5p
miR-548w
miR-3664-3p
miR-5584-5p
miR-6776-5p
miR-4680-3p
miR-718
miR-12132
miR-522-3p
miR-6891-3p
miR-596
miR-3661
miR-6516-5p
miR-3118
miR-4446-3p

miR-122-3p
miR-34a-5p
miR-3615
miR-574-3p
miR-545-3p
miR-6747-3p
miR-654-3p
miR-423-3p
miR-6805-5p
miR-1180-3p
let-7i-3p
miR-425-5p
miR-3619-5p
miR-3138
miR-431-5p
miR-9985
miR-6511b-3p
miR-548b-5p
miR-4448
miR-100-5p
miR-491-5p
miR-23a-3p
miR-3667-3p
miR-1260b
miR-98-3p
miR-320e
miR-6861-3p
miR-7705
miR-7160-5p
miR-199b-5p
miR-424-5p
miR-106b-3p
miR-6798-3p
miR-32-5p
miR-508-3p
miR-141-5p
miR-222-3p
miR-139-5p
miR-5189-5p
miR-96-5p
miR-509-3p
miR-4497
miR-548ag
miR-6762-5p
miR-6764-3p
miR-378g
miR-3202
miR-548az-3p
miR-455-3p
miR-3913-3p
miR-5699-3p
miR-6886-5p
miR-622
miR-4540
miR-5093
miR-4644
miR-4436a
miR-5571-3p
miR-4515
miR-4727-5p
miR-3945
miR-497-3p
miR-6895-3p
miR-4473
miR-6862-3p
miR-4752
miR-514b-3p
miR-6888-3p
miR-4427
miR-12119
miR-3689f
miR-1271-3p
miR-7704
miR-7112-3p
miR-3198
miR-4506
miR-758-5p
miR-5585-3p
miR-363-5p
miR-6827-3p
miR-1236-3p
miR-518e-3p
miR-6727-5p
miR-4633-5p
miR-2115-5p
miR-4712-5p

miR-24-3p	miR-610
miR-148b-5p	miR-204-3p
miR-339-5p	miR-585-3p
miR-323a-3p	miR-345-3p
miR-1248	miR-764
miR-629-3p	miR-1182
miR-450a-5p	miR-3170
miR-18a-5p	miR-6739-5p
miR-3688-3p	miR-3672
miR-99a-5p	miR-19b-2-5p
miR-582-3p	miR-3182
miR-2467-5p	miR-6865-3p
miR-6729-5p	miR-548al
miR-6891-5p	miR-4505
miR-1249-3p	miR-935
miR-183-5p	miR-10395-3p
miR-6832-5p	miR-6759-5p
miR-1268a	miR-6873-5p
miR-204-5p	miR-3173-3p
miR-873-5p	miR-3129-3p
miR-200c-3p	miR-598-5p
miR-22-5p	miR-4747-5p
miR-338-3p	miR-6856-5p
miR-609	miR-6729-3p
miR-197-3p	miR-6769b-3p
miR-454-5p	miR-301b-5p
miR-4504	miR-4660
miR-30d-3p	miR-4798-5p
miR-874-3p	miR-6086
miR-6131	miR-1233-3p
miR-136-5p	miR-6833-5p
miR-342-3p	miR-6770-5p
miR-193a-5p	miR-6740-5p
miR-6734-5p	miR-6773-3p
miR-514a-5p	miR-4735-5p
miR-10b-5p	miR-3192-5p
miR-492	miR-4786-5p
miR-150-5p	miR-4770
miR-6736-5p	miR-512-3p
miR-18b-5p	miR-3911
miR-185-3p	miR-4762-3p
miR-195-5p	miR-548f-3p
let-7c-3p	miR-4433b-3p

miR-10a-5p
miR-6803-3p
miR-4443
miR-500b-5p
miR-589-3p
miR-9-3p
miR-302a-3p
miR-324-3p
miR-1306-3p
miR-885-3p
miR-3157-5p
miR-6511a-3p
miR-140-5p
miR-26b-3p
miR-4684-3p
miR-4707-5p
miR-326
miR-525-3p
miR-301a-5p
miR-920
miR-519c-3p
miR-877-3p
miR-182-5p
miR-6885-5p
miR-576-5p
miR-548h-3p
miR-5010-3p
miR-29a-3p
miR-148a-5p
miR-378i
miR-3064-5p
miR-324-5p
miR-548z
miR-6861-5p
miR-202-5p
miR-3158-3p
miR-95-3p
miR-330-5p
miR-2355-3p
miR-4452
miR-518f-3p
miR-619-5p
miR-4787-5p
miR-6734-3p
miR-3691-5p
miR-379-3p
miR-3939
miR-3177-5p
miR-1269a
miR-3941
miR-3610
miR-4728-3p
miR-549a-5p
miR-494-5p
miR-3942-3p
miR-3151-3p
miR-5694
miR-3183
miR-1271-5p
miR-6820-5p
miR-221-5p
miR-6870-3p
miR-548ba
miR-548at-3p
miR-6744-5p
miR-1277-5p
miR-6892-5p
miR-3974
miR-6737-3p
miR-5585-5p
miR-6880-3p
miR-892c-3p
miR-562
miR-129-2-3p
miR-4755-5p
miR-3928-3p
miR-4637
miR-3131
miR-578
miR-498-5p
miR-6850-5p
miR-4743-3p
miR-1265
miR-3174
miR-4659a-5p
miR-485-3p

miR-499b-5p
miR-3912-3p
miR-331-5p
miR-4503
miR-199a-5p
miR-27a-3p
miR-4804-3p
miR-548at-5p
miR-5003-3p
miR-4326
miR-6754-5p
miR-193b-3p
miR-19b-1-5p
miR-323b-3p
miR-548o-3p
miR-3922-3p
miR-761
miR-6751-5p
miR-374b-3p
miR-4638-5p
miR-149-3p
miR-10400-5p
miR-329-3p
miR-2116-3p
miR-3135a
miR-9983-3p
miR-556-3p
miR-125b-2-3p
miR-6825-5p
miR-2467-3p
miR-17-5p
miR-4508
miR-3657
miR-450b-5p
miR-93-3p
miR-7114-3p
miR-584-3p
miR-365b-3p
miR-449c-5p
miR-33b-5p
miR-365a-3p
miR-5196-5p
miR-3143
miR-152-5p
miR-4653-3p
miR-6755-3p
miR-3688-5p
miR-1264
miR-6769a-3p
miR-4426
miR-549a-3p
miR-6769a-5p
miR-1537-5p
miR-1296-5p
miR-3136-3p
miR-6721-5p
miR-4761-3p
miR-6881-5p
miR-6820-3p
miR-548ar-5p
miR-4667-5p
miR-6505-3p
miR-4781-5p
miR-6882-5p
miR-5191
miR-2276-3p
miR-887-3p
miR-190a-3p
miR-659-5p
miR-675-5p
miR-4450
miR-6741-3p
miR-10522-5p
miR-218-2-3p
miR-6855-5p
miR-656-3p
miR-493-5p
miR-628-5p
miR-4520-5p
miR-8071
miR-548an
miR-1297
miR-4729
miR-299-5p
miR-6076
miR-5090

miR-337-3p
miR-214-5p
miR-11401
miR-4793-3p
miR-377-3p
miR-766-5p
miR-4321
miR-3686
miR-375-3p
miR-6793-5p
miR-496
miR-6840-5p
miR-21-3p
miR-651-3p
miR-6766-3p
miR-509-5p
miR-6728-5p
miR-4757-5p
miR-513a-5p
miR-6129
miR-3117-3p
miR-206
miR-200a-3p
miR-615-5p
miR-1307-5p
miR-134-3p
miR-3200-3p
miR-4659a-3p
miR-99b-3p
miR-518a-3p
miR-499a-5p
miR-297
miR-6745
miR-202-3p
miR-6829-5p
miR-516a-5p
miR-147b-3p
miR-5689
miR-7851-3p
miR-193b-5p
miR-4772-3p
miR-4429
miR-885-5p
miR-6738-5p
miR-7702
miR-3199
miR-376b-3p
miR-320a-5p
miR-7848-3p
miR-329-5p
miR-5193
miR-1249-5p
miR-3155b
miR-101-2-5p
miR-6826-3p
miR-7152-3p
miR-105-3p
miR-6750-3p
miR-4680-5p
miR-656-5p
miR-4284
miR-4291
miR-4783-5p
miR-3689b-3p
miR-4692
miR-6851-3p
miR-4253
miR-7113-5p
miR-7846-3p
miR-8085
miR-6130
miR-4768-5p
miR-548bc
miR-4793-5p
miR-548ab
miR-6768-5p
miR-1912-3p
miR-6845-3p
miR-4635
miR-4640-5p
miR-4677-5p
miR-5696
miR-4476
miR-6772-3p
miR-4652-3p
miR-4796-3p

miR-29a-5p
miR-6737-5p
miR-208b-3p
miR-141-3p
miR-106b-5p
miR-1224-5p
miR-196a-5p
miR-504-5p
miR-10226
miR-765
miR-6836-5p
miR-365a-5p
miR-5701
miR-6757-5p
miR-6124
miR-4451
miR-520a-3p
miR-6743-5p
miR-217-5p
miR-3150b-5p
miR-4467
miR-708-5p
miR-4447
miR-106a-5p
miR-216a-5p
miR-3186-3p
miR-4648
miR-4663
miR-517c-3p

miR-3976
miR-5195-3p
miR-23c
miR-487b-3p
miR-4671-5p
miR-1295b-5p
miR-769-3p
miR-4666a-5p
miR-4444
miR-10399-3p
miR-34c-5p
miR-6514-5p
miR-4802-5p
miR-548a-5p
miR-1208
miR-7156-3p
miR-520d-5p
miR-382-3p
miR-4494
miR-1273c
miR-6818-3p
miR-665
miR-4721
miR-548am-3p
miR-6750-5p
miR-1255b-2-3p
miR-6126
miR-6783-3p
miR-3667-5p
miR-3529-5p
miR-8088
miR-3978
miR-4694-5p
miR-4293
miR-2681-5p
miR-604
miR-6780a-3p
miR-216b-5p
miR-539-3p
miR-3151-5p
miR-8485
miR-548g-3p
miR-550a-3-5p

miR-143-3p
miR-580-3p
miR-3074-5p
miR-200b-5p
miR-4669
miR-3714
miR-6767-3p
miR-518f-5p
miR-4667-3p
miR-875-5p
miR-31-5p
miR-4500
miR-7151-3p
miR-301b-3p
miR-1203
miR-6804-5p
miR-2114-3p
miR-4713-5p
miR-7157-3p
miR-1468-3p
miR-7515
miR-124-5p
miR-4691-3p
miR-7161-5p
miR-137-5p
miR-365b-5p
miR-154-3p
miR-612
miR-5695
miR-4773
miR-6867-5p
miR-4736
miR-6784-5p
miR-374c-3p
miR-3196
miR-424-3p
miR-519a-3p
miR-5707
miR-129-5p
miR-10393-3p
miR-4312
miR-190a-5p
miR-6784-3p

miR-3150b-3p
miR-373-3p
miR-760
miR-6878-3p
miR-1227-3p
miR-3192-3p
miR-548ae-3p
miR-583
miR-3662
miR-4737
miR-4274
miR-6529-3p
miR-4664-5p
miR-4501
miR-3190-5p
miR-557
miR-6813-3p
miR-32-3p
miR-6864-5p
miR-5590-3p
miR-6798-5p
miR-4710
miR-4768-3p
miR-6803-5p
miR-7-5p
miR-6882-3p
miR-942-3p
miR-3908
miR-4520-3p
miR-12125
miR-378j
miR-4636
miR-193a-3p
miR-6783-5p
miR-3146
miR-4766-5p
miR-4489
miR-1283
miR-4640-3p
miR-6868-5p
miR-4789-3p
miR-1269b
miR-196a-3p

miR-544b
miR-369-5p
miR-143-5p
miR-219a-2-3p
miR-7703
miR-6878-5p
miR-6797-3p
miR-643
miR-135a-3p
miR-539-5p
miR-155-3p
miR-520e-5p
miR-3666
miR-3166
miR-5687
miR-548ao-3p
miR-3120-3p
miR-188-3p
miR-3611
miR-671-3p
miR-6077
miR-548a-3p
miR-5187-5p
miR-6831-5p
miR-550a-5p
miR-548h-5p
miR-6842-3p
miR-6513-3p
miR-6716-5p
miR-1287-5p
miR-4662a-5p
miR-186-3p
miR-501-3p
miR-6125
miR-197-5p
miR-3613-3p
miR-660-3p
miR-31-3p
miR-1294
miR-6775-3p
miR-628-3p
miR-6867-3p
miR-8067

miR-5705
miR-487b-5p
miR-7155-5p
miR-4306
miR-6830-3p
miR-6760-5p
miR-2110
miR-128-1-5p
miR-4652-5p
miR-6078
miR-892b
miR-625-3p
miR-495-3p
miR-1292-3p
miR-4659b-5p
miR-7854-3p
miR-4714-3p
miR-181c-3p
miR-4785
miR-128-2-5p
miR-1246
miR-1304-3p
let-7f-5p
miR-4687-3p
miR-6834-5p
miR-4699-5p
miR-924
miR-520c-5p
miR-3133
miR-185-5p
miR-636
miR-374a-3p
miR-548ae-5p
miR-125b-1-3p
miR-28-5p
miR-3119
miR-10525-3p
miR-6796-3p
miR-6744-3p
miR-5584-3p
miR-302d-5p
miR-4462
miR-217-3p

miR-1324
miR-4300
miR-548k
miR-25-5p
miR-4491
miR-499a-3p
miR-130b-3p
miR-602
miR-5010-5p
let-7g-3p
miR-548x-3p
miR-378a-5p
miR-4685-3p
miR-1244
miR-6079
miR-548n
miR-4778-5p
miR-10527-5p
miR-374b-5p
miR-576-3p
miR-5190
miR-7977
miR-7706
miR-3605-5p
miR-3187-3p
miR-1843
miR-218-5p
miR-4800-3p
miR-922
miR-320a-3p
miR-92a-1-5p
miR-6742-3p
miR-6877-3p
miR-4704-5p
miR-6810-3p
miR-10526-3p
miR-181a-3p
miR-629-5p
miR-130a-3p
miR-6852-3p
miR-6846-3p
miR-12122
miR-4453

miR-4802-3p
miR-7974
miR-7153-5p
miR-2117
miR-1252-5p
miR-5002-3p
miR-7154-3p
miR-590-3p
miR-4753-5p
miR-548x-5p
miR-151a-5p
miR-3177-3p
miR-20a-3p
miR-4299
miR-664a-5p
miR-374a-5p
miR-3652
miR-4670-5p
miR-889-3p
miR-548d-3p
miR-5708
miR-4777-3p
miR-4672
miR-1226-3p
miR-574-5p
miR-6780a-5p
miR-505-5p
miR-579-3p
miR-548az-5p
miR-4298
miR-212-5p
miR-7976
miR-626
miR-4320
miR-148b-3p
miR-941
miR-3158-5p
miR-98-5p
miR-10401-3p
miR-4433a-5p
miR-548d-5p
miR-151b
miR-219b-3p

miR-1537-3p
miR-127-3p
miR-192-5p
miR-6746-5p
miR-6780b-5p
miR-551b-5p
miR-486-3p
miR-6790-3p
miR-4273
miR-5704
miR-3606-3p
miR-10395-5p
miR-489-5p
miR-4288
miR-518a-5p
miR-4305
miR-3616-5p
miR-1266-3p
miR-3689c
miR-190b-5p
miR-6069
miR-8059
miR-1302
miR-15b-3p
miR-335-5p
miR-3140-3p
let-7f-1-3p
miR-1179
miR-664b-5p
miR-6859-5p
miR-152-3p
miR-1262
miR-624-5p
miR-4303
miR-648
miR-1282
miR-671-5p
miR-6854-5p
miR-548i
miR-548ay-5p
miR-7849-3p
miR-4677-3p
miR-1255b-5p

miR-6846-5p
miR-340-3p
miR-641
miR-181a-2-3p
let-7b-3p
miR-3157-3p
miR-409-5p
miR-6786-3p
miR-6811-5p
miR-6715b-3p
miR-3167
miR-372-5p
miR-377-5p
miR-6880-5p
miR-3934-5p
miR-4435
miR-33a-3p
miR-301a-3p
miR-96-3p
miR-130b-5p
miR-513c-5p
miR-4432
miR-5579-3p
miR-4475
miR-5691
miR-4803
miR-644a
miR-6785-3p
miR-524-3p
miR-6889-3p
miR-4745-3p
miR-1277-3p
let-7c-5p
miR-142-5p
miR-607
miR-323b-5p
miR-4509
miR-1245b-5p
miR-6839-3p
miR-1470
miR-30c-5p
miR-8061
miR-376a-3p

miR-134-5p
miR-616-3p
miR-877-5p
miR-135a-5p
miR-1238-3p
miR-4707-3p
miR-942-5p
miR-6503-3p
let-7i-5p
miR-425-3p
miR-625-5p
miR-18b-3p
miR-196b-5p
miR-19a-3p
miR-26b-5p
miR-486-5p
miR-3121-5p
miR-484
miR-520a-5p
miR-3926
miR-412-3p
miR-18a-3p
miR-6875-5p
miR-191-5p
miR-6894-5p
miR-6735-5p
miR-548ar-3p
miR-6884-5p
miR-1306-5p
miR-3613-5p
miR-4262
miR-383-3p
miR-6752-3p
miR-4465
miR-6841-5p
miR-9500
miR-6757-3p
miR-7154-5p
miR-6499-5p
miR-582-5p
miR-6849-5p
miR-4732-5p
miR-655-3p

miR-8087
miR-660-5p
miR-6771-5p
miR-5001-3p
miR-188-5p
miR-589-5p
miR-3124-3p
miR-103a-3p
miR-9898
miR-10523-5p
miR-340-5p
miR-3123
miR-3136-5p
miR-6777-3p
miR-590-5p
miR-526a-5p
miR-378a-3p
miR-4795-3p
miR-2277-3p
miR-16-2-3p
miR-3173-5p
miR-145-5p
miR-3127-3p
let-7g-5p
let-7d-5p
miR-6832-3p
miR-210-3p
miR-4482-3p
miR-6746-3p
let-7a-3p
miR-548t-3p
miR-15a-5p
miR-485-5p
miR-6808-3p
miR-26a-5p
miR-4774-5p
miR-521
miR-4272
miR-8070
miR-5685
miR-759
miR-4471
miR-4789-5p

miR-2054
miR-300
miR-670-3p
miR-7159-3p
miR-7159-5p
miR-3649
miR-4329
miR-3605-3p
miR-183-3p
miR-145-3p
miR-5583-5p
miR-613
miR-8062
miR-3713
miR-3529-3p
miR-3147
miR-766-3p
miR-93-5p
miR-101-3p
miR-6866-5p
miR-223-5p
miR-4481
miR-506-5p
miR-3689a-3p
miR-140-3p
miR-6073
miR-181b-5p
miR-30d-5p
let-7a-5p
miR-6851-5p
miR-744-3p
miR-199a-3p
miR-92b-3p
miR-454-3p
miR-199b-3p
miR-107
miR-15b-5p
miR-548e-3p
miR-451b
miR-16-5p
miR-103b
miR-520g-5p
miR-518d-5p

miR-3675-5p
miR-598-3p
miR-548au-5p
miR-6857-5p
miR-1468-5p
miR-567
miR-337-5p
miR-126-3p
miR-20b-5p
miR-144-3p
miR-550a-3p
miR-22-3p
miR-4723-5p
miR-3671
miR-3681-3p
miR-6778-3p
miR-466
miR-5011-3p
miR-563
miR-518d-3p
miR-6781-3p
miR-1268b
miR-431-3p
miR-3141
miR-4418
miR-4267
miR-12124
miR-4472
miR-6801-3p
miR-181a-5p
miR-2355-5p
miR-934
miR-9851-3p
miR-569
miR-510-5p
miR-8080
miR-617
miR-5009-3p
miR-6781-5p
miR-640
miR-554
miR-7158-5p
miR-624-3p

miR-3163
miR-581
miR-3680-3p
miR-296-5p
miR-532-5p
let-7b-5p
miR-548aa
miR-548au-3p
miR-4268
miR-3169
miR-4782-5p
miR-1193
miR-7-1-3p
miR-3936
miR-513c-3p
miR-92a-3p
miR-6507-3p
miR-3658
miR-12133
miR-6890-3p
miR-646
miR-5591-5p
miR-4798-3p
miR-422a
miR-4776-5p
miR-1539
miR-551a
miR-619-3p
miR-3065-3p
miR-6726-3p
miR-19a-5p
miR-1291
miR-4282
miR-3915
miR-2052
miR-411-3p
miR-4690-5p
miR-3606-5p
miR-95-5p
miR-8066
miR-125b-5p
miR-548s
miR-1225-5p

miR-4755-3p
miR-5189-3p
miR-3614-3p
miR-4633-3p
miR-4523
miR-4698
miR-4716-5p
miR-5692c
miR-500a-5p
miR-3194-3p
miR-6858-5p
miR-933
miR-6874-3p
miR-6833-3p
miR-668-5p
miR-4309
miR-4771
miR-371b-3p
miR-6838-3p
miR-4256
miR-8081
miR-4438
miR-3943
miR-614
miR-4666a-3p
miR-218-1-3p
miR-12131
miR-7843-5p
miR-519e-3p
miR-939-5p
miR-7155-3p
miR-4756-5p
miR-4425
miR-4682
miR-1226-5p
miR-595
miR-1827
miR-3189-5p
miR-542-3p
miR-5006-5p
miR-5580-5p
miR-6872-5p
miR-3115

miR-875-3p
miR-642b-3p
miR-503-5p
miR-1255a
miR-708-3p
miR-24-1-5p
miR-3682-3p
miR-506-3p
miR-331-3p
miR-4292
miR-6529-5p
miR-3152-3p
miR-1298-5p
miR-6807-5p
miR-518e-5p
miR-556-5p
miR-548ak
miR-12121
miR-3121-3p
miR-5091
miR-6763-5p
miR-106a-3p
miR-1-3p
miR-544a
miR-7845-5p
miR-4251
miR-4310
miR-1272
miR-4777-5p
miR-3921
miR-208b-5p
miR-4790-3p
miR-1205
miR-5681b
miR-3935
miR-548ao-5p
miR-633
miR-4684-5p
miR-3973
miR-548u
miR-4480
miR-3917
miR-6869-5p

miR-6815-5p
miR-4671-3p
miR-3622b-3p
miR-451a
miR-6792-3p
miR-1915-3p
miR-6859-3p
miR-5582-5p
miR-3059-5p
miR-1912-5p
miR-483-3p
miR-508-5p
miR-147b-5p
miR-519a-5p
miR-4775
miR-4524b-3p
miR-3929
miR-4527
miR-6874-5p
miR-3150a-3p
miR-4655-3p
miR-501-5p
miR-4794
miR-4252
miR-203b-5p
miR-3126-5p
miR-6841-3p
miR-302b-3p
miR-4323
miR-5588-3p
miR-5197-3p
miR-6504-3p
miR-4276
miR-5682
miR-1250-3p
miR-4441
miR-6854-3p
miR-4726-3p
miR-5586-3p
miR-6782-5p
miR-510-3p
miR-891b
miR-373-5p

miR-5008-5p
miR-299-3p
miR-1184
miR-6499-3p
miR-4522
miR-548ai
miR-570-5p
miR-3142
miR-192-3p
miR-5197-5p
miR-6728-3p
miR-586
miR-627-5p
miR-511-5p
miR-3179
miR-4769-5p
miR-6879-3p
miR-4761-5p
miR-222-5p
miR-4528
miR-5087
miR-30b-3p
miR-6868-3p
miR-548ap-5p
miR-10524-5p
miR-8078
miR-6506-3p
miR-4643
miR-5692b
miR-4275
miR-527
miR-7973
miR-3682-5p
miR-4799-3p
miR-3689d
miR-6722-5p
miR-4641
miR-3977
miR-6751-3p
miR-4314
miR-7843-3p
miR-4510
miR-23b-5p

miR-520f-3p
miR-4651
miR-6801-5p
miR-6887-3p
miR-6768-3p
miR-519a-2-5p
miR-520b-5p
miR-4460
miR-11181-5p
miR-132-5p
miR-615-3p
miR-6824-5p
miR-571
miR-6508-5p
miR-4297
miR-4529-3p
miR-6792-5p
miR-215-3p
miR-203a-3p
miR-11181-3p
miR-5571-5p
miR-4778-3p
miR-29b-2-5p
miR-4315
miR-4666b
miR-6774-5p
miR-548ap-3p
miR-302c-5p
miR-328-5p
miR-4650-3p
miR-767-3p
miR-4277
miR-7162-5p
miR-302b-5p
miR-5683
miR-6881-3p
miR-1587
miR-524-5p
miR-5186
miR-4646-5p
miR-6815-3p
miR-3660
miR-8054

miR-1251-3p
miR-548bb-3p
miR-4436b-5p
miR-4693-5p
miR-5007-3p
miR-6771-3p
miR-519b-5p
miR-519c-5p
miR-522-5p
miR-523-5p
miR-7111-3p
miR-3195
miR-520f-5p
miR-4325
miR-4700-5p
miR-3184-3p
miR-552-5p
miR-652-3p
miR-122b-3p
miR-4746-5p
miR-3668
miR-384
miR-4529-5p
miR-7158-3p
miR-449b-3p
miR-519e-5p
miR-4801
miR-1825
miR-2909
miR-4711-3p
miR-873-3p
miR-4289
miR-6716-3p
miR-5188
miR-6776-3p
miR-3684
miR-2682-3p
miR-205-3p
miR-4533
miR-4780
miR-1976
miR-4769-3p
miR-378e

miR-592
miR-630
miR-6500-5p
miR-4711-5p
miR-8084
miR-1185-1-3p
miR-6764-5p
miR-606
miR-4485-5p
miR-593-3p
miR-8053
miR-939-3p
miR-7151-5p
miR-491-3p
miR-1305
miR-4632-3p
miR-650
miR-4744
miR-8064
miR-302a-5p
miR-3201
miR-10396b-3p
miR-4486
miR-4759
miR-937-3p
miR-30c-2-3p
miR-187-3p
miR-4645-3p
miR-4733-3p
miR-711
miR-3154
miR-1251-5p
miR-591
miR-3618
miR-4278
miR-6738-3p
miR-6080
miR-6873-3p
miR-8052
miR-4675
miR-3153
miR-6736-3p
miR-4294

miR-938
miR-499b-3p
miR-6814-3p
miR-4431
miR-1243
miR-135b-3p
miR-3927-3p
miR-6853-5p
miR-548m
miR-4727-3p
miR-3691-3p
miR-8083
miR-662
miR-3651
miR-647
miR-6759-3p
miR-205-5p
miR-8065
miR-4254
miR-216a-3p
miR-12114
miR-5094
miR-3934-3p
miR-5587-3p
miR-219a-1-3p
miR-513a-3p
let-7a-2-3p
miR-4764-3p
miR-645
miR-4322
miR-4772-5p
miR-6779-3p
miR-4539
miR-433-5p
miR-488-3p
miR-12126
miR-449c-3p
miR-1206
miR-642a-5p
miR-4739
miR-1343-3p
miR-638
miR-519d-5p

miR-3674
miR-3126-3p
miR-1238-5p
miR-1204
miR-4495
miR-4760-5p
miR-3670
miR-137-3p
miR-3190-3p
miR-6775-5p
miR-298
miR-4285
miR-6889-5p
miR-514a-3p
miR-8074
miR-580-5p
miR-147a
miR-3616-3p
miR-3924
miR-3655
miR-937-5p
miR-12128
miR-4428
miR-3178
miR-6778-5p
miR-1228-5p
miR-4717-5p
miR-4520-2-3p
miR-575
miR-224-3p
miR-4492
miR-4781-3p
miR-552-3p
miR-519b-3p
miR-5004-5p
miR-4646-3p
miR-620
miR-6892-3p
miR-4258
miR-550b-2-5p
miR-2116-5p
miR-4507
miR-892c-5p

miR-507
miR-7110-5p
miR-138-5p
miR-890
miR-4653-5p
miR-516b-3p
miR-3117-5p
miR-3975
miR-4756-3p
miR-1229-5p
miR-12117
miR-4750-5p
miR-1207-3p
miR-611
miR-4668-5p
miR-338-5p
miR-5582-3p
miR-488-5p
miR-4697-5p
miR-2113
miR-181b-2-3p
miR-3680-5p
miR-664b-3p
miR-6748-5p
miR-5579-5p
miR-940
miR-2682-5p
miR-4498
miR-1288-5p
miR-5583-3p
miR-4324
miR-9718
miR-4661-5p
miR-4774-3p
miR-6895-5p
miR-520b-3p
miR-639
miR-6848-3p
miR-6845-5p
miR-20b-3p
miR-4440
miR-6501-3p
miR-8068

miR-1304-5p
miR-6743-3p
miR-432-3p
miR-4751
miR-4513
miR-3620-5p
miR-5047
miR-3186-5p
miR-8056
miR-6862-5p
miR-3134
miR-1321
miR-4804-5p
miR-4738-3p
miR-4316
miR-4654
miR-6857-3p
miR-9851-5p
miR-7109-3p
miR-874-5p
miR-1914-3p
miR-618
miR-7108-5p
miR-658
miR-4287
miR-4704-3p
miR-10396a-3p
miR-3919
miR-1257
miR-4699-3p
miR-4269
miR-374c-5p
miR-34a-3p
miR-99a-3p
miR-6515-5p
miR-548v
miR-603
miR-593-5p
miR-520e-3p
miR-6724-5p
miR-371b-5p
miR-3188
miR-3191-3p

miR-1207-5p
miR-579-5p
miR-1275
miR-6502-3p
miR-1258
miR-4760-3p
miR-6883-3p
miR-8082
miR-6835-3p
miR-4255
miR-105-5p
miR-548av-3p
miR-4639-5p
miR-652-5p
miR-2114-5p
miR-325
miR-4517
miR-548bb-5p
miR-4649-3p
miR-296-3p
miR-4676-3p
miR-3925-5p
miR-4632-5p
miR-888-5p
miR-10393-5p
miR-371a-3p
miR-888-3p
miR-585-5p
miR-6808-5p
miR-668-3p
miR-6819-5p
miR-6715b-5p
miR-383-5p
miR-6887-5p
miR-1225-3p
miR-6779-5p
miR-129-1-3p
miR-4730
miR-4749-3p
miR-6504-5p
miR-3925-3p
miR-12130
miR-4754

miR-3181
miR-4731-5p
miR-5586-5p
miR-6730-3p
miR-378f
miR-10392-5p
miR-6770-3p
miR-675-3p
miR-1233-5p
miR-15a-3p
miR-1263
miR-190b-3p
miR-4537
miR-6758-3p
miR-4499
miR-10394-3p
miR-4279
miR-5590-5p
miR-564
miR-3654
miR-1273h-5p
miR-367-3p
miR-642b-5p
miR-6812-5p
miR-3132
miR-548ax
miR-6509-3p
miR-548ac
miR-6827-5p
miR-9986
miR-5088-3p
miR-944
miR-6836-3p
miR-195-3p
miR-635
miR-6829-3p
miR-10394-5p
miR-7112-5p
miR-1199-3p
miR-7110-3p
miR-1538
miR-8055
miR-6795-3p

miR-5591-3p
miR-7850-5p
miR-103a-2-5p
miR-6068
miR-5092
miR-133b
miR-4662b
miR-378b
miR-548t-5p
miR-500b-3p
miR-548ad-3p
miR-513b-5p
miR-655-5p
miR-3918
miR-6758-5p
miR-3165
miR-876-5p
miR-6756-3p
miR-6742-5p
miR-4686
miR-6071
miR-511-3p
miR-26a-2-3p
miR-1234-3p
miR-587
miR-631
miR-4776-3p
miR-4308
miR-5002-5p
miR-6806-3p
miR-6839-5p
miR-5589-5p
miR-6719-3p
miR-4295
miR-452-3p
miR-8060
miR-12120
miR-548aj-3p
miR-6754-3p
miR-6749-3p
miR-1252-3p
miR-4318
miR-4304

miR-6717-5p
miR-1178-5p
miR-4676-5p
miR-10392-3p
miR-194-3p
miR-599
miR-4283
miR-4328
miR-891a-5p
miR-6866-3p
miR-4714-5p
miR-5699-5p
miR-4668-3p
miR-3675-3p
miR-6756-5p
miR-6828-5p
miR-6766-5p
miR-6885-3p
miR-3916
miR-4661-3p
miR-654-5p
miR-187-5p
miR-3116
let-7f-2-3p
miR-24-2-5p
miR-4270
miR-1224-3p
miR-548aw
miR-520g-3p
miR-3148
miR-7978
miR-5787
miR-4690-3p
miR-512-5p
miR-4743-5p
miR-6823-5p
miR-1289
miR-627-3p
miR-3162-5p
miR-1245a
miR-6735-3p
miR-4763-5p
miR-559

miR-5089-5p
miR-6847-5p
miR-3162-3p
miR-198
miR-5739
miR-6748-3p
miR-4742-5p
miR-3160-5p
miR-1228-3p
miR-6088
miR-516a-3p
miR-498-3p
miR-4717-3p
miR-6807-3p
miR-4479
miR-3161
miR-4795-5p
miR-3685
miR-4259
miR-6838-5p
miR-518c-3p
miR-4423-5p
miR-6511b-5p
miR-1909-5p
miR-200a-5p
miR-6128
miR-4519
miR-1181
miR-770-5p
miR-3156-3p
miR-450b-3p
miR-4753-3p
miR-4474-5p
miR-4302
miR-10400-3p
miR-4634
miR-4725-3p
miR-1245b-3p
miR-3922-5p
miR-3176
miR-4695-5p
miR-520c-3p
miR-6876-3p

miR-1178-3p
miR-4767
miR-3689a-5p
miR-3689b-5p
miR-4455
miR-637
miR-4695-3p
miR-3940-5p
miR-3683
miR-4521
miR-12123
miR-3920
miR-4705
miR-1296-3p
miR-9900
miR-4437
miR-1197
miR-4457
miR-6844
miR-6511a-5p
miR-4763-3p
miR-4696
miR-6514-3p
miR-1288-3p
miR-5011-5p
miR-448
miR-4713-3p
miR-4530
miR-6510-3p
miR-10401-5p
miR-6816-3p
miR-6720-5p
miR-6872-3p
miR-3650
miR-380-3p
miR-6884-3p
miR-6816-5p
miR-3194-5p
miR-302c-3p
miR-8058
miR-4317
miR-8057
miR-4458

miR-553
miR-6503-5p
miR-634
miR-3689e
miR-1911-3p
miR-6782-3p
miR-3614-5p
miR-212-3p
miR-663b
miR-12129
miR-6772-5p
miR-7844-5p
miR-6787-5p
miR-4797-5p
miR-4456
miR-3074-3p
miR-758-3p
miR-6133
miR-6733-3p
miR-3663-5p
miR-4281
miR-12116
miR-4700-3p
miR-4423-3p
miR-2276-5p
miR-526b-5p
miR-4797-3p
miR-4722-5p
miR-5706
miR-4650-5p
miR-6793-3p
miR-6760-3p
miR-4678
miR-6821-5p
miR-3677-5p
miR-3677-3p
miR-5680
miR-6834-3p
miR-4439
miR-6814-5p
miR-376a-2-5p
miR-3937
miR-2861

miR-1202
miR-4709-5p
miR-4327
miR-6894-3p
let-7e-3p
miR-4420
miR-4477a
miR-6165
miR-4720-5p
miR-8072
miR-4683
miR-876-3p
miR-6823-3p
miR-7157-5p
miR-6762-3p
miR-4731-3p
miR-541-5p
miR-130a-5p
miR-661
miR-4691-5p
miR-5589-3p
miR-4664-3p
miR-138-2-3p
miR-6747-5p
miR-5006-3p
miR-4694-3p
miR-6835-5p
miR-10396b-5p
miR-1913
miR-4706
miR-4716-3p
miR-936
miR-6718-5p
miR-7161-3p
miR-3144-3p
miR-6796-5p
miR-526b-3p
miR-1276
miR-3944-5p
miR-8079
miR-4266
miR-149-5p
miR-5700

miR-1915-5p
miR-550b-3p
miR-10397-3p
miR-3679-3p
miR-6888-5p
miR-6715a-3p
miR-10396a-5p
miR-6863
miR-103a-1-5p
miR-4722-3p
miR-526a-3p
miR-4280
miR-4734
miR-3646
miR-1247-3p
miR-4799-5p
miR-520h
miR-6127
miR-555
miR-133a-5p
miR-92a-2-5p
miR-5581-5p
miR-4464
miR-4311
miR-1185-2-3p
miR-3144-5p
miR-4260
miR-4708-5p
miR-4445-3p
miR-6870-5p
miR-1908-3p
miR-375-5p
miR-3621
miR-5581-3p
miR-138-1-3p
miR-3145-3p
miR-4740-3p
miR-4485-3p
miR-4724-3p
miR-3085-3p
miR-4697-3p
miR-1199-5p
miR-608

miR-2277-5p
miR-6858-3p
miR-4685-5p
miR-8077
miR-6749-5p
miR-6791-3p
miR-6732-5p
miR-3692-5p
miR-4483
miR-6871-5p
miR-621
miR-6822-5p
miR-7975
miR-3160-3p
miR-4490
miR-9902
miR-3972
miR-10398-5p
miR-6826-5p
miR-4673
miR-1200
miR-5572
miR-3622a-5p
miR-4757-3p
miR-6837-5p
miR-572
miR-4470
miR-3692-3p
miR-6761-5p
miR-6512-5p
miR-4478
miR-3678-5p
miR-1295b-3p
miR-4319
miR-5702
miR-2115-3p
miR-4746-3p
miR-4723-3p
miR-6752-5p
miR-5008-3p
miR-5194
miR-3145-5p
miR-3130-3p

miR-6886-3p
miR-487a-5p
miR-4738-5p
miR-4487
miR-6789-3p
miR-6893-5p
miR-6800-3p
miR-4758-3p
miR-3152-5p
miR-200c-5p
miR-4442
miR-4296
miR-1227-5p
miR-4445-5p
miR-4728-5p
miR-4665-3p
miR-1293
miR-381-5p
miR-1287-3p
miR-5681a
miR-5003-5p
miR-6830-5p
miR-12118
miR-34b-5p
miR-4446-5p
miR-663a
miR-3191-5p
miR-11399
miR-4758-5p
miR-649
miR-6821-3p
miR-4787-3p
miR-7107-3p
miR-4762-5p
miR-519d-3p
miR-4512
miR-6081
miR-12113
miR-4307
miR-6840-3p
miR-1185-5p
miR-1267
miR-153-5p

miR-601
miR-8073
miR-4764-5p
miR-208a-3p
miR-6070
miR-3944-3p
miR-6811-3p
miR-6824-3p
miR-1231
miR-4750-3p
miR-6806-5p
miR-6822-3p
miR-4703-5p
miR-6084
miR-5089-3p
miR-3197
miR-3159
miR-7150
miR-4796-5p
miR-573
miR-3928-5p
miR-4261
miR-7109-5p
miR-3663-3p
miR-3923
miR-4674
miR-6875-3p
miR-3150a-5p
miR-504-3p
miR-7108-3p
miR-6788-3p
miR-1973
miR-1180-5p
miR-6809-3p
miR-3085-5p
miR-4749-5p
miR-7152-5p
miR-9899
miR-3648
miR-3064-3p
miR-3185
miR-4740-5p
miR-3622a-3p

miR-6733-5p
miR-889-5p
miR-4693-3p
miR-4645-5p
miR-6075
miR-6799-3p
miR-4725-5p
miR-12115
miR-1910-5p
miR-514b-5p
miR-208a-5p
miR-5703
miR-5004-3p
miR-1253
miR-203a-5p
miR-216b-3p
