# Supplementary material for: Clues for Improving the Pathophysiology Knowledge for Endometriosis Using Plasma Micro-RNA Expression
Source: Diagnostics (Basel). 2022 Jan 12;12(1):175. doi: 10.3390/diagnostics12010175 (PMC8774370; doi:10.3390/diagnostics12010175)
Supplement: Supplementary file 1 [file diagnostics-12-00175-s001.zip › Table S2.pdf]

Table S2. Accuracy metrics of the top 57 miRNAs

miRNAs	AUC	Sensitivity	Specificity	Accuracy	Regulation
miR-6502-5p	0.603	0.292	0.913	0.435	UP
miR-515-5p	0.604	0.273	0.935	0.425	UP
miR-548j-5p	0.628	0.474	0.783	0.545	UP
miR-29b-1-5p	0.68	0.708	0.652	0.695	UP
miR-4748	0.605	0.623	0.587	0.615	UP
miR-548p	0.628	0.604	0.652	0.615	-
miR-5697	0.607	0.649	0.565	0.63	-
miR-3124-5p	0.604	0.338	0.87	0.46	-
miR-4999-5p	0.612	0.506	0.717	0.555	-
miR-6501-5p	0.6	0.331	0.87	0.455	-
miR-1270	0.613	0.487	0.739	0.545	-
miR-433-3p	0.605	0.558	0.652	0.58	-
miR-548ah-3p	0.619	0.565	0.674	0.59	-
miR-1278	0.612	0.701	0.522	0.66	-
miR-548l	0.654	0.591	0.717	0.62	-
miR-4511	0.624	0.682	0.565	0.655	-
miR-3940-3p	0.604	0.708	0.5	0.66	-
miR-5009-5p	0.616	0.688	0.543	0.655	-
miR-10399-5p	0.601	0.442	0.761	0.515	-
miR-1292-5p	0.61	0.721	0.5	0.67	-
miR-144-5p	0.603	0.468	0.739	0.53	-
miR-3942-5p	0.6	0.526	0.674	0.56	-
miR-92b-5p	0.6	0.526	0.674	0.56	-
miR-362-5p	0.607	0.344	0.87	0.465	-
miR-1285-3p	0.604	0.338	0.87	0.46	-
miR-3913-5p	0.635	0.552	0.717	0.59	-
miR-548q	0.607	0.584	0.63	0.595	-
miR-30e-3p	0.627	0.429	0.826	0.52	-
miR-151a-3p	0.606	0.539	0.674	0.57	-
miR-4732-3p	0.62	0.675	0.565	0.65	-
miR-421	0.618	0.649	0.587	0.635	-
miR-6789-5p	0.604	0.513	0.696	0.555	-
miR-27b-5p	0.606	0.734	0.478	0.675	-
miR-1910-3p	0.603	0.727	0.478	0.67	-
miR-6773-5p	0.607	0.584	0.63	0.595	-
miR-542-5p	0.619	0.89	0.348	0.765	-
miR-548f-5p	0.604	0.643	0.565	0.625	-
miR-1250-5p	0.602	0.682	0.522	0.645	-
miR-1972	0.619	0.63	0.609	0.625	-
miR-548ay-3p	0.603	0.662	0.543	0.635	-
miR-4466	0.602	0.357	0.848	0.47	-

miR-6785-5p	0.6	0.831	0.37	0.725	-
miR-6777-5p	0.611	0.396	0.826	0.495	-
miR-4514	0.604	0.578	0.63	0.59	-
miR-6802-5p	0.608	0.695	0.522	0.655	-
miR-4658	0.625	0.773	0.478	0.705	-
miR-124-3p	0.656	0.747	0.565	0.705	-
miR-4655-5p	0.604	0.448	0.761	0.52	-
miR-1343-5p	0.611	0.831	0.391	0.73	-
miR-1266-5p	0.601	0.766	0.435	0.69	-
miR-548b-3p	0.614	0.727	0.5	0.675	-
miR-6509-5p	0.606	0.734	0.478	0.675	-
miR-7107-5p	0.612	0.506	0.717	0.555	-
miR-8089	0.6	0.656	0.543	0.63	-
miR-6813-5p	0.622	0.701	0.543	0.665	-
miR-3137	0.617	0.734	0.5	0.68	DOWN
miR-3168	0.618	0.779	0.457	0.705	DOWN
