# Supplementary material for: Clues for Improving the Pathophysiology Knowledge for Endometriosis Using Plasma Micro-RNA Expression
Source: Diagnostics (Basel). 2022 Jan 12;12(1):175. doi: 10.3390/diagnostics12010175 (PMC8774370; doi:10.3390/diagnostics12010175)
Supplement: Supplementary file 1 [file diagnostics-12-00175-s001.zip › Table S3.pdf]

Table S3. Specific analysis of hsa-miR-124-3p.

**miRNAs**	**Ad/Inv**	**Prolif**	**Apop**	**Angio**	**Inf**	**EMR**	**Met / Mig**	**Immune -resp / esc**	**Neuro**	**LTP**	**Other**	**Targets / Regulator**	**Pathways**
hsa-miR-124-3p	X	X	X	X	X	-	X	-	X		Regulates cell proliferation and invasion of ectopic endometrium ; EMT ; promotes trophoblast cells pyroptosis ; Chemosensitivity ; enhanced the migration and epithelial-stromal transformation of endometrial stromal cells extracted from eutopic endometrium in subjects with adenomyosis, bone formation	ITGB3, PDE4B, EGR2, CRKL, p65, ABCA2, MGAT5, DNA methyltransferase 3B, EDNRB, DAPK1, T – synthase, FIP200, TRIM14	mTor, STAT3, PI3K / AKT, NF – KB, ERK, PLGF / ROS, FGF2 – EGFR, MAPK, GSK3B / Beta catenine

Ad/Inv: Adhesion / Invasion , Prolif : Proliferation ; Apop: Apoptosis ; Angio: Angiogenesis ; Inf: Inflammation ; EMR : Extracellular Matrix Remodeling ; Met / Mig : Metastasis and Migration ; Immune Resp / esc : Immune Response or escape ; Neuro f : Neurogenic function ; LTP: Link to Pollutants
