# Supplementary material for: Clues for Improving the Pathophysiology Knowledge for Endometriosis Using Plasma Micro-RNA Expression
Source: Diagnostics (Basel). 2022 Jan 12;12(1):175. doi: 10.3390/diagnostics12010175 (PMC8774370; doi:10.3390/diagnostics12010175)
Supplement: Supplementary file 1 [file diagnostics-12-00175-s001.zip › Table S4.pdf]

Table S4. miRNA-associated benign and malignant disorders.

mirRNAs	Benign disorders	Malignant disorders
miR-4999-5p	-	Colorectal cancer
miR-6501-5p	Differentially expressed in COVID 19 patients	
miR-1270		Bladder cancer, Thyroid cancer, Osteosarcoma, ovarian cancer
miR-433-3p		Oral squamous cell carcinoma, Esophageal squamous cell carcinoma, glioma, gastric cancer
miR-548ah-3p	Urinary miRNA associated with diabetic nephropathy	
miR-1278		Papillary cancer, lung cancer, ovarian cancer
miR-1292-5p		Gastric cancer
miR-144-5p	Renal injury, retinal degeneration, sickle cell disease, depressive disorders, premature ovarian failure	Lung cancer, glioblastoma, Bladder cancer
miR-362-5p		Oral squamous cell carcinoma, lung cancer, breast cancer, hepatocellular carcinoma, bladder cancer, leukemia, neuroblastoma
miR-1285-3p	Atrial fibrillation, Nicotine initiation and addiction	Hepatocellular carcinoma, osteosarcoma, colorectal cancer
miR-548q	Biomarker of response to weight loss	
miR-151a-3p	Arthritis, Neurodegenerative disorders, ischemic stroke, keratoconus,azthenozoospermy,	Nasopharyngeal carcinoma, cervical cancer, renal cancer

miR-421	Epilepsy, Vitiligo, spinal cord injury	Nasopharyngeal carcinoma, NSCLC, breast cancer, gastric cancer, neuroendocrine prostate cancer, cervical cancer
miR-27b-5p		Gastric cancer, ovarian cancer
miR-1910-3p	Psoriasis, endometrial receptivity	Colorectal cancer, breast cancer
miR-542-5p	Diabetic retinopathy, myocardial injury	Osteosarcoma, breast cancer, gastric cancer, colorectal cancer
miR-548f-5p		
miR-1250-5p		Non-Hodgkin's lymphoma
miR-1972	Early onset preeclampsia, atrial fibrillation, hepatic steatosis, urinary level to predict diabetic nephropathy	Osteosarcoma, laryngeal squamous cell carcinoma,
miR-548ay-3p	Circadian rhythm	
miR-6785-5p		Lung carcinoma, Gastric carcinoma
miR-6777-5p		Lung cancer
miR-4514	Associated with intracerebral hemorrhage	
miR-4658	Sensitive to intracerebral hemorrhage	
miR-1266-5p	Skin diseases,	Acute lymphoblastic leukemia, breast cancer, hepatocellular carcinoma, prostate cancer
miR-548b-3p		Breast cancer, lung cancer

	miR-6509-5p		Hepatocellular carcinoma
	miR-7107-5p	Obesity, Febrile seizures	
