# Supplementary material for: Clues for Improving the Pathophysiology Knowledge for Endometriosis Using Plasma Micro-RNA Expression
Source: Diagnostics (Basel). 2022 Jan 12;12(1):175. doi: 10.3390/diagnostics12010175 (PMC8774370; doi:10.3390/diagnostics12010175)
Supplement: Supplementary file 1 [file diagnostics-12-00175-s001.zip › Table S5.pdf]

Table S5. miRNA-associated pathophysiologic pathways.

mirRNAs	Ad/ Inv	Prolif	Apopt	Angio	Inf	EMR	Met / Mig	Immune Resp / escT	Neuro f	LTP	Ster / Horm	Therap sens	Other
miR-1266-5p	X	X	X	-	-	-	X	-	-	-	X		
miR-548b-3p	-	X	X	-	-	-	X	-	-	-			-
miR-6509-5p	X	X	-	-	-	-	X	-	-	-			-
miR-7107-5p	-	-	-	-	-	-	-	-	-	-			
miR-151a-3p	X	X	-	X	-	-	X	X	-	-			Postmenopausal osteoporosis
miR-421	-	X	X	-	X	-	-	-	X	X		X	Regulating human melanocyte survival
miR-27b-5p	-	X	X	-	-	-	X	-	-	-			-
miR-1910-3p	-	X	-	-	-	-	X	-	-	-			Autophagy / Involved in endometrial receptivity in PCOS women
miR-542-5p	X	X	X			X	X			X			Inhibits Hyperglycemia and Hyperlipoidemia, inhibit mitochondrial and cytoplasmic protein synthesis, Suppresses EMT of trophoblast, Autophagy

miR-548f-5p	-	-	-	-	-	-	-	-	-	-		Smooth muscle alpha actin expression
miR-1250-5p	-	X	-	-	-	-	X	-	-	-		-
miR-1972	X	X	X	X			X	X			X	
miR-548ay-3p	-	-	-	-	-	-	-	-	-	-		
miR-6785-5p	X	X	X	X			X				X	
miR-6777-5p												
miR-4514												
miR-548q	-	-	-	-	-	-	-	-	-	-		
miR-1292-5p	X	X	-	-	-	-	X	-	-	-		-
miR-144-5p	X	X	X	X			X	X			X	
miR-362-5p	X	X	X	-	-	-	X	X	-	-	X	Proliferation and inhibits apoptosis of trophoblast cells
miR-1285-3p	X	X	X	-	-	-	-	-	-	-		
miR-4999-5p	-	-	-	-	-	-	-	-	-	-		Reprograms glucose metabolism
miR-6501-5p [71]												

miR-1270	X	X	X				X	X				X	
miR-433-3p	X	X	-	-	-	-	X	-	-	-		X	Bone formation regulator
miR-548ah-3p													
miR-1278	X	X			X		X					X	Autophagy, Vitamin D

Ad/Inv: Adhesion / Invasion , Prolif : Proliferation ; Apopt: Apoptosis ; Angio: Angiogenesis ; Inf: Inflammation ; EMR : Extracellular Matrix Remodeling ; Met / Mig : Metastasis and Migration ; Immune Resp / esc : Immune Response or escape ; Neuro f : Neurogenic function ; LTP:
